# Role and mechanism of gut microbiota and metabolites in schizophrenia complicated with sleep disorder

**DOI:** 10.1080/19490976.2025.2607817

**Published:** 2025-12-29

**Authors:** Ziqi Huang, Zixuan Huang, Zhiqiang Du, Xuezheng Gao, Ying Jiang, Zhenhe Zhou, Haohao Zhu

**Affiliations:** a Wuxi Mental Health Center, Wannan Medical College Graduate Training Unit, Wuxi, People's Republic of China; b Affiliated Mental Health Center of Jiangnan University, Wuxi, People's Republic of China

**Keywords:** Schizophrenia, sleep disorder, gut microbiota, metabolites, microbiota–gut–brain axis

## Abstract

Schizophrenia (SCZ) is a major mental disorder with a high disability rate, and its pathogenesis involves the interaction of multiple factors such as genetics, environment, immunity, and neurodevelopment. Most SCZ patients are complicated with significant sleep disorder (SD), manifested as insomnia, sleep fragmentation, reduction in slow-wave sleep, and circadian rhythm disturbance. This comorbidity not only aggravates the severity of psychiatric symptoms but also significantly impacts treatment adherence and long-term prognosis. In recent years, the role of gut microbiota and its metabolites in mental diseases has received increasing attention. Existing studies have shown that the gut microbiota regulates brain function through the microbiota–gut–brain axis, affects the metabolism of neurotransmitters and immune-inflammatory responses, and thus may play an important role in the occurrence and development of SCZ and SD. However, the specific mechanism is still not clear enough at present, and there are still deficiencies in relevant studies. This article reviews the characteristics of gut microbiota diversity and metabolome related to sleep in SCZ patients, explores the potential mechanism of the role of gut microbiota and its metabolites in SCZ complicated with SD, provides a new microbial-metabolic perspective for understanding the pathogenesis of SD in SCZ patients, and suggests the potential therapeutic value of improving sleep problems in SCZ patients through probiotic intervention or metabolic regulation. These findings not only deepen the understanding of the comorbidity mechanism of mental diseases and SD but also provide a theoretical basis for new intervention strategies based on the gut–brain axis.

## 1. Introduction

Schizophrenia (SCZ) is a severe mental disorder characterized by positive symptoms such as delusions and hallucinations, negative symptoms like loss of motivation and social withdrawal, as well as cognitive symptoms including deficits in working memory and cognitive flexibility.^1^ In addition, the comorbidity of sleep disorder (SD) is extremely common in SCZ patients. These patients often experience difficulties falling asleep, sleep maintenance problems, disrupted sleep architecture, and circadian rhythm dysregulation, which severely impact their quality of life, social functioning, and long-term disease prognosis.^2^ Cross-sectional studies indicate that approximately four out of five individuals with psychosis suffer from at least one type of SD, with an average of 3.3 types of SD per patient. This deterioration in sleep quality can further exacerbate psychiatric symptoms, leading to more frequent paranoid ideation, hallucinatory experiences, thought disorganization, and higher levels of depression and anxiety.^3^ Studies have found that SCZ patients with impaired sleep quality and circadian rhythm disruption exhibit higher scores on the negative symptom dimension of clinical assessment scales and more pronounced cognitive dysfunction.^4^ Furthermore, not only does SD worsen the psychotic symptoms of SCZ, but the core symptoms of SCZ also disrupt sleep physiology. Polysomnography studies have revealed that more severe positive symptoms are associated with shorter rapid eye movement (REM) latency, longer sleep onset latency, and lower sleep efficiency; whereas prominent negative symptoms are linked to reduced slow-wave amplitude in non-rapid eye movement (NREM) sleep and shorter REM onset latency.^5^ These findings suggest a bidirectional pathological cycle between SCZ and SD, indicating that SD is not merely a common comorbidity of SCZ but also a key factor influencing its symptom expression and disease progression.

The gut microbiota refers to the complex microbial community colonizing the human gastrointestinal tract, including bacteria, archaea, fungi, viruses, and protists, among which bacteria dominate.^6^ It is estimated that the number of microorganisms in the human gut is as high as 10^14^, nearly 1.3 times the total number of human cells, and the number of their genomes far exceeds that of the human genome. Therefore, it is called the “second genome” of the human body.^7^ The gut microbiota and the host influence each other, jointly adapt to the environment, and affect nutrition, immunity, and disease susceptibility. These microorganisms conduct two-way communication with the central nervous system (CNS) through four main pathways: the nervous system, the endocrine system, the immune system, and the metabolic system.^8^ Patients with mental disorders exhibit a decrease in butyrate-producing bacteria, an increase in lactate-producing bacteria, and an increase in bacteria related to glutamate and gamma-aminobutyric acid (GABA) metabolism.^9^ It is noteworthy that in SCZ patients, this specific gut microbiota disruption may contribute to the development of both psychiatric symptoms and sleep problems simultaneously through mechanisms such as affecting the balance of neurotransmitters, promoting neuroinflammation, and interfering with circadian rhythms.

Currently, there is still a lack of systematic summaries regarding the specific mechanism of the role of gut microbiota and its metabolites in SCZ combined with SD. In this review, we integrate existing evidence. Firstly, we introduce the characteristics of the gut microbiota in SCZ and SD patients. By comparing these results, we analyze the common influences of key microbiota and metabolites in SCZ and SD and explore the mechanisms through which the gut microbiota exerts its effects via pathways such as neuroimmunity, the endocrine system, and neurotransmitters. Finally, we propose the potential application of intervention strategies targeting the gut microbiota in improving SCZ combined with SD, providing a theoretical basis for the development of precision treatment strategies based on the microbiome.

## 2. Clinical association between SCZ and SD

### 2.1. Epidemiological characteristics of SD in SCZ patients

In addition to the typical psychiatric symptoms, SD, as one of the most common comorbidities of SCZ, has received increasing clinical attention in recent years. Studies have shown that the prevalence of SD among SCZ patients is significantly higher than that in the general population, affecting their quality of life and disease prognosis. By observing the differences in sleep architecture between SCZ patients and healthy controls through sleep electroencephalogram (EEG), it was found that the REM latency of SCZ patients decreased and the REM density increased, and the percentage of REM sleep was closely related to the severity of symptoms.^10^ During NREM sleep, reductions in slow-wave sleep, decreases in sleep spindle wave density, reductions in amplitude, and shortenings in duration were detected in SCZ patients.^11^ Meanwhile, SD exhibits different characteristics among different groups of SCZ patients. For example, there are significant differences between hospitalized patients and outpatients in terms of sleep time, activity level, and the stability of the biological clock, and the manifestations of SD in hospitalized patients are more severe.^12^ This indicates that the severity of the clinical symptoms of SCZ depends not only on the pathological mechanism itself but also on the combined influence of multiple factors such as the living environment and treatment status of patients.

### 2.2 Influence of SD on SCZ symptoms

SCZ patients have sleep problems such as low sleep efficiency and poor sleep quality. Poor sleep at night further leads to daytime dysfunction and a decline in quality of life. A large number of clinical studies have confirmed that there is a significant and specific association pattern between SD and the core symptom groups of SCZ patients. SD not only aggravates the positive symptoms of SCZ (such as hallucinations and delusions) but also exacerbates the negative symptoms (such as emotional apathy and social withdrawal), which in turn affect patients' cognitive function.^13^ Moreover, SCZ patients with SD exhibit poor treatment compliance, akathisia, and aggressive speech.^14^ In an eight-year longitudinal study, utilizing Kaplan–Meier survival analysis combined with the log-rank test, it was revealed that patients with schizophrenia-spectrum disorders who experienced frequent insomnia had a significantly higher cumulative risk of suicidal behavior, which confirmed that SD increase the risk of attempted suicide in these patients.^15^ These results show the negative impacts on SCZ patients due to sleep problems and emphasize the necessity of strengthening clinical attention to the sleep of SCZ patients.

### 2.3 Limitations of existing treatments for SCZ complicated with SD

Currently, the main method for treating patients with SCZ combined with SD is to use traditional antipsychotic drugs. Among them, atypical antipsychotic drugs, such as clozapine, olanzapine, quetiapine, risperidone, ziprasidone, and paliperidone, can all help with the total sleep time and sleep efficiency of SCZ patients.^16^ However, the efficacy of these medications in improving SD is limited, and they are accompanied by a range of side effects, such as weight gain and metabolic syndrome.^17^ Even some patients still experience problems such as poor sleep quality and sleep architecture disorders after receiving antipsychotic drug treatment.^18^

In clinical practice, although drug treatment is the main intervention measure, patients' concerns about drug dependence and side effects make some patients reluctant to use these drugs for a long time. In addition, cognitive behavioral therapy (CBT) for insomnia has shown certain effectiveness in improving the sleep of patients with persistent delusions or hallucinations, but more research support is still needed for its clinical application.^19^ It can be seen that existing treatment methods face multiple limitations when dealing with patients with SCZ combined with SD, and there is an urgent need to explore new treatment targets and methods. For example, in recent years, studies on melatonin have shown its efficacy in regulating the sleep–wake rhythm, improving sleep quality, and ameliorating SCZ symptoms.^20^ Besides, biofeedback therapy and other nondrug intervention methods, such as light therapy and sleep hygiene education, are also considered effective adjuvant treatment modalities that can improve patients' sleep quality.^21^

Overall, the existing treatment methods for patients with SCZ combined with SD have obvious limitations in terms of efficacy and tolerability. There is an urgent need to explore new treatment regimens through further research and clinical trials, with the expectation of providing more effective and safe treatment options for patients.

## 3. Characteristics of gut microbiota in patients with SCZ complicated with SD

### 3.1 Gut microbiota dysbiosis in SCZ patients

Multiple studies have found that the gut microbiota has intricate relationships with the pathological mechanism, symptom manifestations, and cognitive function of SCZ (Table 1). The results of analyzing the composition of the gut microbiota using high-throughput sequencing technology show that compared with the healthy control group, the richness index (Chao) and diversity index (Shannon) of the gut microbiota in SCZ patients are both lower, and there are also differences in the composition and abundance of specific microbiota. A between-group comparison of the relative abundance of gut microbiota using the Mann‒Whitney U test revealed a significant decrease in Bacteroidetes, a phylum known for its important anti-inflammatory role in immune responses, alongside a significant increase in Proteobacteria. At the genus level, the abundances of *Faecalibacterium*, *Coprococcus*, and *Bacteroides*, which are related to the production of butyric acid with anti-inflammatory and neuroprotective effects, are decreased, while the abundances of Prevotella and Collinsella are increased. The reduction in bacteria that produce short-chain fatty acids (SCFAs) and the increase in proinflammatory bacteria may be related to the severity of the condition of SCZ patients.^22-24^ In studies on different stages of the disease, acute-phase SCZ patients show a decrease in *Haemophilus* and *Faecalibacterium*, while remitted SCZ patients are characterized by an increase in Megamonas and Megasphaera.^25^ In addition, oral resident bacteria such as *Veillonella atypica*, *Streptococcus salivarius*, and *Bifidobacterium dentium* are significantly enriched in the gut microbiota of SCZ patients. This pathological state may weaken the intestinal barrier and immune defense functions, creating favorable conditions for the colonization of oral bacteria in the gut.^26^ A comprehensive multikingdom microbiome analysis encompassing bacteria, fungi, archaea, and viruses found that the gut of SCZ patients exhibited an increase in microbial taxa such as *Streptococcus* and *Desulfovibrio* (which utilize amino acids or fatty acids as carbon sources) and the gut archaeon Methanobrevibacter smithii, alongside a confirmed decrease in butyrate-producing bacteria.^27^ Overall, alterations in the gut microbiota of SCZ patients are primarily characterized by an imbalance of specific bacterial taxa, abnormal colonization of oral bacteria, and a reduction in beneficial bacteria. These multikingdom microbial changes suggest a potential link between gut dysbiosis and neuroinflammation in SCZ patients.

**Table 1.** Studies on the changes in the gut microbiota of SCZ patients.

Author and year of publication	Models	Species	Microbiota profiling methods	Sample size	Dysbiosis of microbiota
Li et al. [28]	SCZ	Humans	16S rRNA sequencing	SCZ: n = 82 HC: n = 80	*Succinivibrio*↑ *Corynebacterium*↓
Zhu et al. [26]	SCZ	Humans	Metagenomic shotgun sequencing	SCZ: n = 90 HC: n = 81	facultative anaerobes↑ oral resident bacteria↑
Gubert et al. [29]	Metabotropic glutamate receptor 5 knockout mouse model of SCZ	Male mGlu5 KO mice	16S rRNA sequencing	WT: n = 6 KO: n = 6	*Erysipelotrichaceae*↓ *Allobaculum*↓
Li et al. [30]	SCZ	Humans	16S rRNA sequencing	SCZ: n = 38 HC: n = 38	*Ruminococcus*↓ *Roseburia*↓ *Veillonella*↑
Zhu et al. [25]	Acute and remission SCZ	Humans	16S rRNA sequencing	Acute SCZ: n = 42 Remission SCZ: n = 40 HC: n = 44	Remission: *Megamonas*↑ *Megasphaera*↑ Acute: *Haemophilus*↓ *Faecalibacterium*↓
Deng et al. [31]	Aggressive and non-aggressive SCZ	Humans	16S rRNA sequencing	SCZ-Ag: n = 25 NSCZ-Ag: n = 25	SCZ-Ag: *α* diversity and *β* diversity↓*Bacteroides*↓ *Faecalibacterium*↓ Prevotella↑
Gokulakrishnan et al. [32]	Drug-naïve and risperidone-treated SCZ	Humans	16S rRNA sequencing	DN SCZ: n = 20 RISP SCZ: n = 20 HC: n = 20	RISP SCZ: *Ruminococcus*↑ *Bifidobacterium*: HCs→DN SCZ→RISP SCZ↓
Wang et al. [33]	SCZ	Humans	16S rRNA and metagenomic shotgun sequencing	SCZ: n = 29 HC: n = 30	*Megasphaera*↑ *Clostridium*↑
Wang et al. [34]	SCZ	Humans	Metagenomic shotgun sequencing	SCZ: n = 127 HC: n = 92	Pro-inflammatory species↑: *Bacteroides_ovatus*; *Bacteroides_stercoris*; *Lactobacillus_ruminis* SCFA generation reduction and anti-inflammatory species↓: *Bifidobacterium_longum*; *Roseburia_faecis*
Zhu et al. [35]	SCZ with different BMI levels	Humans	16S rRNA sequencing	OW-SCZ: n = 88 NW-SCZ: n = 68 OW-NC: n = 48 NW-NC: n = 108	SCZ-OW enriched with Roseburia, Fusobacterium, Enterococcus SCZ-NW enriched with Synergistes, Methanobrevibacter

Abbreviations: SCZ: schizophrenia; HC: healthy control; WT: wild type; mGlu5 KO: metabotropic glutamate receptor 5 knockout mice; SCZ-Ag: schizophrenia with aggression; NSCZ-Ag: schizophrenia without aggression; DN: grug-naïve; RISP: risperidone-treated; OW: overweight/obese; NW: normal weight.

### 3.2 Microbiota changes related to SD

Similar results have also been observed in human and animal models with SDs (Table 2). In studies on the sleep of healthy adults, it was found that the diversity of the microbiota was positively correlated with sleep efficiency and total sleep time and negatively correlated with sleep fragmentation. In short, the diversity of the gut microbiota promotes healthier sleep.^36^ In addition, another study conducted among preschool children also showed that children with relatively longer sleep time at night had different gut microbiota structure compared with those with shorter sleep time at night. The study also found that *Bifidobacterium* and *Bacteroides* in the microbiota were associated with longer sleep time at night, higher sleep efficiency, and shorter wake time at night among preschool children, respectively.^37^ A study indicated that both sleep and circadian rhythm disruptions lead to gut microbiota dysbiosis in both humans and animal models, characterized by an increase in pathogenic bacteria and a decrease in beneficial bacteria. In humans, insufficient and poor-quality sleep is often associated with an increase in Prevotellaceae and Erysipelotrichaceae and a decrease in Ruminococcus. In animal models, sleep deprivation and fragmentation result in an increase in Lachnospiraceae and Erysipelotrichaceae and a decrease in Lactobacillaceae and Bifidobacteriaceae.^38^ Parallel experiments in humans and mice have revealed consistent circadian oscillations in the gut microbiota across species. Furthermore, transplanting the gut microbiota from humans before and after experiencing jet lag into germ-free mice resulted in altered microbiota profiles and the emergence of metabolic abnormalities such as weight gain, elevated blood sugar, and body fat accumulation.^39^ These findings demonstrate the significant translational potential from animal models to human studies, although this translation process must also carefully consider the limitations imposed by species-specific differences.

**Table 2.** Studies on the changes in the gut microbiota in SD.

Author and year of Publication	Models	Species	Microbiota profiling methods	Sample size	Dysbiosis of microbiota
Poroyko et al. [40]	Sleep fragmentation	Male C57BL/6J mice	16S rRNA sequencing	SF: n = 30 NS: n = 30	*Lachnospiraceae*↑ *Ruminococcaceae*↑
Grosicki et al. [41]	PSQI for sleep measuring	Humans	16S rRNA sequencing	GS: n = 19 PS: n = 9	Prevotella was positively correlated with the PSQI score
Liu et al. [42]	Acute sleep–wake cycle shift	Humans	16S rRNA sequencing	*n* = 22	F/B↑
Li et al. [43]	Acute and chronic insomnia	Humans	16S rRNA sequencing	AID: n = 20 CID: n = 38 HC: n = 38	CID: *Actinobacteria*↑ *Faecalibacterium*↓ AID: *Firmicutes↓ Bacteroide*) ↑
Haimov et al. [44]	Insomnia	Humans	16S rRNA sequencing	*n* = 72	*Lachnoclostridium*↓
Yang et al. [45]	72 h REM sleep stage deprivation	Male C57BL/6J mice	16S rRNA sequencing	SD: n = 10 NS: n = 10	*Lactobacillaceae*↓ *Ruminococcus*↓ *Akkermansia↓ Enterobacter*↑
Gao et al. [46]	24 h sleep deprivation	Humans	16S rRNA sequencing	*n* = 11	F/B ratio↑ Butyrate↓
Huang et al. [47]	RBD	Humans	16S rRNA sequencing	Early PD: n = 36 RBD: n = 170 RBD-FDR: n = 127 HC: n = 108	PRD: Butyrate-producing bacteria↓ Collinsella↑ *Desulfovibrio*↑ RBD-FDR: *Eubacterium*↓
Seong et al. [48]	PSQI for sleep measuring	Humans	16S rRNA sequencing	GS: n = 99 PS: n = 60	PS: *Faecalibacterium*↓ *Prevotella*↓

Abbreviations: SF: sleep fragmentation; NS: normal sleep; F/B: firmicutes/bacteroidetes ratio; AID: acute insomnia disorder; CID: chronic insomnia disorder; HC: healthy control; SD: sleep deprivation; RBD: rapid eye movement sleep behavior disorder; FDR: first-degree relatives; PD: Parkinson's disease; PSQI: Pittsburgh Sleep Quality Index; GS: PSQI score ≤ 5; PS: PSQI score > 5.

Furthermore, patients with different types of SD appear to exhibit distinct gut microbiota profiles. Those with chronic insomnia disorder primarily show an increased abundance of Actinobacteria and a reduced abundance of the anti-inflammatory genus *Faecalibacterium* – a microbial shift potentially linked to a long-term low-grade inflammatory state. In contrast, patients with acute insomnia disorder exhibit a gut microbiota characterized by a decrease in Firmicutes and an increase in Bacteroides.^43^ This pattern closely mirrors findings from acute 24-hour sleep deprivation experiments,^46^ suggesting that such short-term microbial imbalance may represent a stress response to abrupt changes in sleep patterns. Similarly, study has also demonstrated an increase in Firmicutes and a decrease in Bacteroidetes, leading to a higher Firmicutes/Bacteroidetes (F/B) ratio.^49^ Research on acute sleep schedule delay further confirmed that alterations in the sleep‒wake cycle increase the F/B ratio compared to baseline.^42^ The study also reported that acute circadian disruption promotes disease-associated purine metabolism and butyrate-generating acetyl-CoA fermentation pathways, which are related to host energy metabolism and inflammatory responses. Notably, these metabolic changes tended to reverse during the recovery period.

### 3.3 Microbiota commonalities in SCZ complicated with SD

Based on previous studies, we have found that both SCZ patients and individuals with SD exhibit a reduction in gut microbiota diversity, accompanied by similar changes in the composition of specific microbiota. This lack of diversity may undermine the stability of the gut microecosystem, thereby increasing the risk of disease. In terms of microbiota composition, a decrease in anti-inflammatory bacteria and an increase in proinflammatory bacteria have been observed, which are closely related to disease susceptibility. In addition, colonic organisms, such as *Bacteroides*, *Bifidobacterium*, and *Faecalibacterium*, ferment carbohydrates that escape proximal digestion and indigestible oligosaccharides, resulting in the synthesis of SCFAs. A reduction in these genera significantly affects the production of butyrate, propionate, acetate, etc.^50^. Meanwhile, it has also been found that the abundances of specific microbiota closely related to sleep are decreased in SCZ patients. For example, the decrease in *Bifidobacterium* is associated with reduced sleep efficiency and sleep fragmentation, while the increase in Prevotella may further disrupt the sleep structure. In a study investigating the relationship between sleep and gut microbiota composition in psychiatric disorders, the Mann‒Whitney U test revealed that *Ellagibacter isourolithinifaciens* and *Senegalimassilia faecalis* were significantly enriched in patients with good sleep quality. At the genus level, Senegalimassilia was positively correlated with better sleep quality in patients.^51^ The aforementioned characteristics of microbial alterations indicate that SCZ and SD interact synergistically through disruptions in the microbiota–gut–brain axis (MGBA). This process involves mechanisms such as reduced production of short-chain fatty acids (SCFAs), enhanced systemic inflammation, and circadian rhythm disruption, all of which contribute to and exacerbate the co-occurrence and progression of these two conditions.

## 4. Potential mechanisms of gut microbiota in SCZ complicated with SD

**Figure 1.**
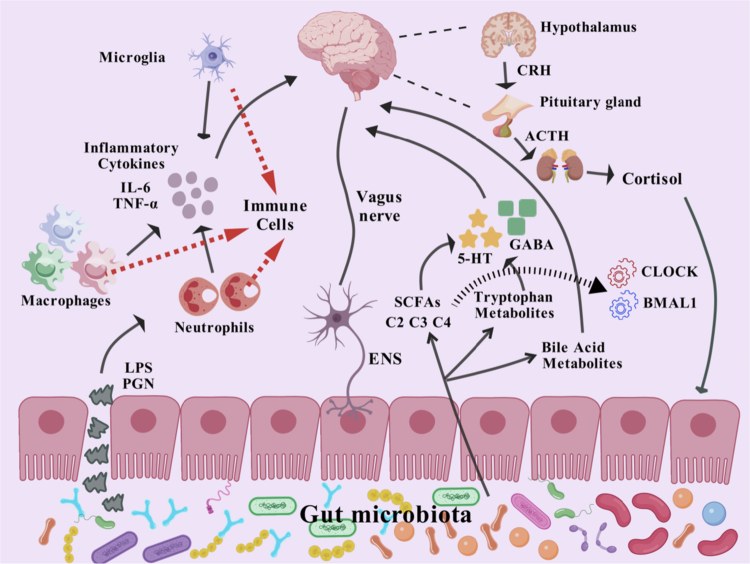
Pathways of interaction between gut microbiota and their metabolites via the gut–brain axis. The gut microbiota and its metabolites profoundly influence the function of the CNS through four major pathways, namely, the vagus nerve, the endocrine system, the immune system, and the metabolic system, forming a complex regulatory network of the brain‒gut axis. From the perspective of neuroendocrine regulation, the gut microbiota affects cortisol release by regulating the HPA axis. Cortisol, as the core regulatory hormone for the body's stress response, is not only regulated by the microbiota but also has a feedback regulatory effect on the composition and function of the microbiota by directly acting on glucocorticoid receptors of intestinal epithelial cells and indirectly changing the gut microenvironment. At the molecular mechanism level, SCFAs produced by the metabolism of gut microbiota, as key signaling molecules, can not only reach the brain through the blood circulation to enhance the integrity of the blood‒brain barrier but also regulate the synthesis and release of neurotransmitters together with tryptophan metabolites. Meanwhile, the diurnal fluctuations of these metabolites are closely related to the host's biological clock and influence the host's circadian rhythm by regulating the expression of core clock genes such as BMAL1 and CLOCK. In addition, substances such as lipopolysaccharides released when the microbiota structure is imbalanced can activate immune cells and prompt them to secrete proinflammatory factors such as interleukin-6 (IL-6) and tumor necrosis factor-*α* (TNF-*α*). These inflammatory mediators may induce neuroinflammatory responses after spreading to the central nervous system through the circulatory system. The gut microbiota can also form real-time communication with the CNS through the vagus nerve, which is like a “two-way highway”. Moreover, the brain can also act on the microbiota in reverse by regulating intestinal peristalsis, barrier function, and the living environment of the microbiota. This precise two-way regulatory mechanism jointly participates in and influences the operation of complex brain functions such as mood regulation, stress response, and higher cognitive functions. This figure was drawn using BioGDP.com.^55^

Within the complex pathological network through which gut microbiota influences mental disorders, the host's genetic background and environmental factors collectively form a critical foundation for disease susceptibility. A genome-wide pleiotropic analysis has revealed extensive genetic correlations between gastrointestinal diseases and psychiatric disorders, shared pathogenic genes and pathways, as well as their genetic associations with gut microbiota, confirming the pivotal role of the MGBA in the shared genetic basis of these two disease categories.^52^ Various environmental factors, such as geographic location, dietary patterns, and lifestyle, dynamically regulate the richness and diversity of the gut microbiota, and are also important elements influencing the functional homeostasis of the MGBA.^53^ The MGBA, as a key regulatory hub for host‒microbe interactions, integrates the functional coupling between the gut microecosystem and the CNS through a multilevel two-way communication network involving the nervous, endocrine, and immune systems (Figure 1). Specifically, the gut microbiota can not only directly regulate the activity of the CNS through the synaptic transmission of the vagus nerve but also influence the permeability of the blood–brain barrier, neuroinflammatory responses, and the synthesis and metabolism of neurotransmitters via cytokine-mediated immune regulatory pathways.^54^ Dysfunction of the MGBA can lead to pathological changes such as abnormal activation of the hypothalamus–pituitary–adrenal (HPA) axis and continuous activation of microglia, which are then closely related to the comorbid mechanisms of neuropsychiatric diseases such as SCZ and SD.

### 4.1 Neurotransmitter regulation

In the studies of SCZ and SD, abnormal metabolism of neurotransmitters is considered an important factor affecting disease symptoms, and gut microbiota and its metabolites play a key role in the production and functional regulation of neurotransmitters.^56^ For example, abnormal metabolism of neurotransmitters such as serotonin (5-HT), GABA, and dopamine (DA) can interfere with neurotransmission in SCZ and sleep regulation in SD.^57^ Studies have found that gut microbiota directly affects the biosynthesis of 5-HT by regulating the tryptophan metabolic pathway. In the pineal gland, 5-HT undergoes a sequential enzymatic conversion process. Through *N*-acetylation, it is transformed into *N*-acetylserotonin, which then undergoes O-methylation to synthesize melatonin. The integrity of this biosynthetic pathway directly influences the production level of endogenous melatonin, thereby affecting the initiation and maintenance of sleep.^58^^,^^59^. Therefore, gut microbiota can regulate this metabolic pathway by influencing the availability of tryptophan, increasing the conversion of tryptophan to 5-HT rather than the kynurenine pathway through specific microbiota, thus increasing the levels of 5-HT and melatonin. It has been found that these specific microbiota such as *Lactobacillus* and *Bifidobacterium*, can not only promote the conversion of tryptophan to 5-HT but also directly synthesize GABA.^60^ GABA, as the main inhibitory neurotransmitter in the CNS, is closely associated with anxiety and depressive symptoms in SCZ patients due to its metabolic disorders. By acting on GABA receptors in the CNS, it can effectively reduce nerve excitability and prolong slow-wave sleep time.^61^ In summary, metabolic disorders of neurotransmitters play an important role in the pathological mechanisms of SCZ and SD. The gut microbiota, by influencing neurotransmitters, not only affects psychiatric symptoms but also exacerbate the condition by influencing sleep. Therefore, intervention measures targeting neurotransmitter metabolism provide new ideas and strategies for the treatment of patients with SCZ combined with SD.

### 4.2 Immune-inflammatory pathway

Changes in the composition of gut microbiota can promote the occurrence of neuroinflammation, and this inflammatory response is regarded as one of the important pathological mechanisms of multiple mental disorders, including SCZ and SD. Gut microbiota and its metabolites are key molecules that regulate the maturation, morphology, and function of microglia. Changes in gut microbiota can activate glial cells, leading to the release of proinflammatory cytokines such as IL-6 and TNF-*α*. These factors not only affect the survival and function of neurons but also disrupt the integrity of the blood‒brain barrier, facilitating the infiltration of peripheral inflammatory cytokines and neuroactive metabolites into the CNS, and ultimately inducing neuroinflammatory responses.^62^^,^^63^ Longitudinal design studies on SCZ patients have shown that higher levels of IL-6 and TNF-*α* are associated with decreased levels of brain-derived neurotrophic factor (BDNF) and cognitive dysfunction. SCZ patients with higher levels of inflammatory markers and lower levels of BDNF are more likely to experience symptom deterioration over time.^64^ The immune system also play a role in specific symptom areas. Inflammatory stimuli can change the neural activity in the ventral striatum area of healthy individuals in a way similar to that of SCZ patients, resulting in reduced motivation and deficits in reward processing, indicating that inflammation may be a pathophysiological pathway affecting negative and cognitive symptoms.^65^ Studies have found that SD is associated with increases in inflammatory markers such as C-reactive protein (CRP) and IL-6. An acute increase in inflammatory markers can change the amount and depth of sleep.^66^ Statistical analysis using the Mann‒Whitney U test revealed that, compared to the control group, mice subjected to 72 hours of REM sleep deprivation exhibited elevated plasma LPS concentrations and a significant upregulation of TNF-*α* levels.^45^ After restricting sleep in rats, it was found that the selective filtering function of the blood‒brain barrier was weakened, reducing its ability to block harmful substances and exacerbating the risk of neuroinflammation.^67^ Clinical interventions have shown that after CBT was used to treat elderly patients with chronic and primary insomnia, a reduction in markers of inflammatory risk was observed, demonstrating a significant association between the immune-inflammatory pathway and SD.^68^ In terms of treatment, anti-inflammatory treatment may be an important strategy for improving patients with SCZ combined with SD. By targeting the inflammatory pathway to reduce the activation level of microglia, the production of proinflammatory cytokines can be reduced, thereby improving the overall symptoms and quality of life of patients.^69^ Future studies should focus on exploring the precise treatment effects and potential neuroprotective mechanisms of specific anti-inflammatory intervention methods (such as cytokine antagonists or microbiome regulation) on SCZ combined with SD.

### 4.3 Neuroendocrine regulation

The HPA axis, as a core component of the neuroendocrine system, plays a crucial role in the regulation of stress responses, and its dysfunction has become one of the important mechanisms by which the gut microbiota mediates SD.^70^ Under stress conditions, corticotropin-releasing factor (CRF) secreted by the hypothalamus activates the anterior pituitary to release adrenocorticotropic hormone (ACTH), which then stimulates the adrenal cortex to synthesize glucocorticoids, such as cortisol.^71^ Long-term excessive exposure to glucocorticoids can affect neuronal plasticity in different regions of the brain. These structural remodels are closely related to cognitive dysfunction and increased anxiety-like behaviors in animal experiments, suggesting that glucocorticoid-mediated abnormal neural circuits may be an important pathological basis for cognitive deficits and affective symptoms in chronic stress and mental disorders.^72^ A large amount of clinical evidence shows that HPA axis dysfunction is prevalent in SCZ. Specifically, the cortisol awakening response (CAR) of SCZ patients presents an obvious abnormal pattern, including excessive hyperactivity or dull attenuation. More characteristically, the morning basal cortisol levels of these patients are significantly higher than those of healthy people. And this pattern of HPA axis dysfunction is clearly disease-specific, and similar changes have not been observed in high-risk psychiatric populations.^73^ Neuroimaging studies consistently show that the pineal gland of SCZ patients has characteristic volume reduction and increased calcification, and this morphological abnormality directly leads to impaired synthesis and secretion functions of melatonin.^74^ This multilevel neuroendocrine disorder is correlated with the severity of clinical symptoms of SCZ. Meanwhile, it also disrupts the sleep‒wake cycle, resulting in an extended sleep onset latency and disrupted sleep continuity. It can be seen that neuroendocrine abnormalities and SD form a complex two-way regulatory network in the course of SCZ. Sleep deficiency activates hypothalamic CRF neurons, leading to excessive activation of the HPA axis and increased cortisol secretion. High cortisol levels, in turn, inhibit melatonin synthesis and interfere with the sleep regulatory center, further aggravating SD. This disorder forms a pathological closed loop of “endocrine disorder – sleep problem – disease deterioration”, which participates in the progression of SCZ and the maintenance of symptoms.

### 4.4 Metabolite-mediated regulation

Metabolites such as SCFAs, tryptophan derivatives, and bile acids produced by gut microbiota are directly or indirectly involved in the regulation of the CNS, affecting the balance of neurotransmitters, maintaining immune homeostasis, and regulating circadian rhythms. Specifically, dysbiosis can disrupt the integrity of the intestinal barrier, increase intestinal permeability, and simultaneously impair the function of the blood‒brain barrier. The disruption of these dual barriers makes it easier for harmful substances such as bacterial metabolites and proinflammatory cytokines to enter the CNS and trigger neuroinflammatory responses.^75^ Among them, butyrate, as a key metabolite of the microbiota, is not only crucial for maintaining the structural integrity of the intestinal mucosa,^76^ but also can cross the blood‒brain barrier to regulate the sleep‒wake cycle by activating the vagus nerve pathway. Animal experiments have confirmed that intravenous injection of butyrate can significantly increase the NREMS time of rats.^77^ Butyrate not only plays an important role in sleep regulation, but also the increase in serum butyrate levels is positively correlated with the reduction in the Positive and Negative Syndrome Scale (PANSS) positive symptom subscale scores of SCZ patients,^78^ suggesting its potential neuroprotective effect. Besides abnormal butyrate metabolism, SCZ patients also exhibit significant tryptophan metabolism disorders. Studies have found that the plasma tryptophan levels in SCZ patients are generally reduced.^79^ This lower tryptophan level may be attributed to the enhanced conversion of tryptophan metabolism to the kynurenine pathway and the reduced conversion to the 5-HT synthesis pathway.^80^ This shift in the metabolic pathway leads to a reduction in the synthesis of the neurotransmitter 5-HT and the neuromodulator melatonin. Since these substances play a key role in sleep regulation, their deficiency exacerbate SDs in SCZ patients by affecting sleep onset and disrupting the sleep–wake rhythm.^81^ In studies on metabolite enrichment analysis, it was found that the concentrations of popular metabolites in the bile acid pathway of SCZ patients, such as glycocholic acid, taurodeoxycholic acid, and taurochenodeoxycholic acid, were significantly reduced.^82^ The decrease in bile acid levels can promote the formation of a chronic low-grade inflammatory microenvironment by affecting anti-inflammatory signaling pathways, thereby increasing the risk of developing various brain diseases such as neurodegenerative diseases and mental disorders.^83^ Associations between chronic insomnia patients and the structure and composition of specific bile acids have also been found.^84^ Therefore, this abnormality in the metabolic axis is not only a characteristic biomarker of SCZ but also one of the key mechanisms for the onset of SD in SCZ. Interventions targeting gut microbiota and its metabolites can provide new therapeutic targets for improving patients with SCZ combined with SD.

### 4.5 Circadian rhythm disturbance

There exists a close relationship between gut microbiota and circadian rhythms. The composition of gut microbiota fluctuates in response to changes in the host's central biological clock, while disruption of the gut microbiota can also affect the central clock, as well as intestinal immune function and nutrient metabolism.^85^ The composition and metabolites of gut microbiota have a unique regulatory effect on the expression of host clock genes (such as Clock and Bmal1). Studies have found that microbial metabolites can regulate or change the biological rhythms of the central nervous system and the liver. For example, SCFAs can directly regulate the expression of clock genes in liver cells, thereby affecting the stability of the host's biological rhythm.^86^ Gut microbiota imbalance is prevalent among SCZ patients, and this imbalance state can affect the expression of host clock genes through the gut–brain axis. Studies have confirmed that by analyzing the mononuclear blood cells of first-episode SCZ patients, it was found that compared with the healthy control group, the expression levels of CLOCK, PER2, and CRY1 genes in the patients' blood cells were significantly reduced. In fibroblasts obtained from chronic SCZ patients, the rhythmic expression of clock genes CRY1 and PER2 was lost.^87^ These studies reveal the interaction between gut microbiota through its metabolites and clock genes and play an important role in the stability of the circadian rhythm of SCZ patients. More importantly, alterations in the expression of clock genes not only impact the structure of the gut microbiota but also further affect the efficiency of core circadian rhythm output pathways. Studies have indicated a significant interaction between circadian rhythm disruption and the MGBA, which continuously influences the health status and clinical symptom presentation of patients throughout the course of mental illness.^88^ Integrating the content discussed earlier in this review, the circadian clock regulates physiological processes such as sleep, metabolism, and immunity by modulating neuroendocrine activities and autonomic nervous system activity. The failure of this output pathway can lead to disrupted sleep architecture, dysregulated rhythmicity of stress hormones, and periodic disturbances in metabolic and immune responses, which collectively form the clinical symptomatic basis commonly observed in SCZ patients. Meanwhile, changes in the expression of clock genes will, in turn, affect the composition of gut microbiota, showing a reduction in richness and diversity, forming a complex interaction network.^89^ For instance, studies have found that the core activating gene (Bmal1) and inhibitory genes (Per1, Per2) of the host circadian clock collectively regulate the diurnal oscillations of gut microbiota composition. Gene knockout of these clock components abolished these rhythmic fluctuations and altered the microbial community structure in mice.^90^ In-depth research on the relationship between gut microbiota and its metabolites and clock genes will not only help to understand the pathogenesis of SCZ but also provide new ideas for future treatment strategies based on gut microbiota intervention. For example, by adjusting the diet or supplementing with probiotics to improve the composition of gut microbiota, and regulate the expression of clock genes to optimize the transmission efficiency of circadian rhythm signals and improve the clinical symptoms of SCZ patients.

## 5. Potential treatment strategies based on gut microbiota dysbiosis in SCZ complicated with SD

**Figure 2.**
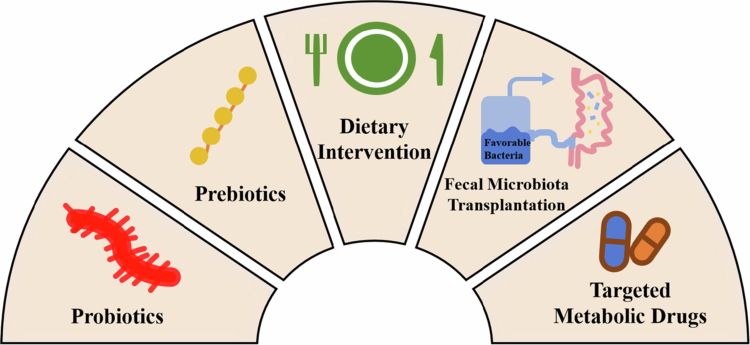
Microbiota-targeted intervention programs for improving sleep in SCZ patients. This schematic diagram presents five treatment strategies targeting the gut microbiota, including probiotics, prebiotics, dietary interventions, FMT, and targeted metabolite drug treatments. These treatment methods mainly improve symptoms by regulating the functional pathways of the brain–gut–microbe axis, providing new treatment options for sleep problems accompanied by mental disorders such as SCZ.

Although SD has been confirmed to be closely related to the occurrence and development of multiple mental disorders, the current intervention studies targeting SCZ combined with SD are still insufficient. Most of the existing treatment strategies focus on symptom control, such as the improvement of core psychiatric symptoms by antipsychotic drugs, while rarely paying attention to the underlying gut microbiota dysbiosis mechanism. Based on this, the following discusses new treatment strategies targeting the gut microecology, including specific probiotics, prebiotics, dietary interventions, fecal microbiota transplantation (FMT), and targeted metabolite treatments, aiming to improve sleep problems and related psychiatric symptoms in SCZ patients by regulating the function of the gut‒brain axis (Figure 2).

### 5.1 Probiotics

Differences in gut microbiota among individuals can affect the efficacy and toxicity of drugs. Therefore, personalized gut microbiota regulation can help improve drug responses.^91^ By detecting the gut microbiota of SCZ patients, personalized probiotic regimens can be formulated to effectively adjust the gut microbiota composition and thus play a role in improving symptoms. The efficacy of probiotics varies by strain. In terms of inflammation and stress regulation, different strains exhibit distinct mechanisms of action. For example, *Bifidobacterium* infantis35624 has been shown to reverse HPA axis dysfunction, an effect associated with the normalization of the anti-inflammatory to pro-inflammatory cytokine ratio, which may be clinically relevant.^92^ Lactobacillus rhamnosus (JB-1) has been demonstrated to reduce stress-induced corticosterone levels and alleviate anxiety- and depression-like behaviors.^93^ *Bifidobacterium* longum NCC3001 (BL) mediates signaling through the vagus nerve pathway within the MGBA, normalizing anxiety-like behaviors and hippocampal BDNF levels in mice with low-grade intestinal inflammation.^94^^,^^95^ This strain has also demonstrated antidepressant effects in human trials, which are related to changes in the activities of multiple brain regions involved in emotion processing.^96^ In a double-blind study utilizing miniature polysomnography to record sleep in patients with insomnia, participants were administered either Lactobacillus plantarumPS128 or a placebo. The results indicated that the PS128 group exhibited improvements in fatigue levels, alterations in brain wave activity, and a reduction in the number of awakenings during deep sleep. Additionally, symptoms of anxiety and depression were alleviated. This suggests that specific probiotic strains may modulate sleep architecture and emotional state, potentially through mechanisms involving the microbiota‒gut‒brain axis, such as regulation of the GABAergic system.^97^ Regarding multistrain probiotic interventions, a double-blind, randomized, placebo-controlled trial involving patients with bipolar disorder and schizophrenia-spectrum disorders demonstrated that supplementation with a multistrain probiotic formula improved biomarkers related to gut permeability and inflammation, while also exerting positive effects on cognitive function.^98^ These research results reveal the potential therapeutic value of probiotic supplements in the regulation of neurological functions and provide an important theoretical basis for further exploring the intervention mechanisms of probiotics in the field of neural regulation.

### 5.2 Prebiotics

Prebiotics refer to a class of functional dietary components that can resist the action of host digestive enzymes. They play physiological regulatory functions by selectively promoting the metabolic activities of beneficial gut microbiota.^98^ In multiple trials, galactooligosaccharides, inulin-type fructans, and their synbiotic preparations have been found to reduce the levels of proinflammatory factors such as high-sensitivity CRP, IL-6, and TNF-*α*, indicating that specific prebiotic interventions can regulate the expression of inflammation-related biomarkers.^99^ Inulin, as a soluble dietary fiber, can improve the psychiatric and behavioral symptoms of SCZ model mice. Meanwhile, an increase in the abundance of beneficial bacteria and an improvement in intestinal permeability have also been observed in the mice.^100^ Taking a compound preparation of fructooligosaccharides and galactooligosaccharides also improved the intestinal function of SCZ model mice.^101^ By remodeling the composition of gut microbiota, reducing the levels of proinflammatory cytokines, and enhancing intestinal permeability to reduce the contact between pathogens and the intestinal mucus layer, this may be an important pathophysiological basis for improving the core symptoms of multiple mental disorders. Another study has shown that the combined intervention of short-chain galactooligosaccharides and long-chain fructooligosaccharides significantly promotes the production of acetate and butyrate among SCFAs. These prebiotic components can also effectively regulate the expression levels of the core clock genes BMAL1 and CLOCK in the hypothalamus and hippocampus.^102^ These results reveal that prebiotics play a neuromodulatory function through the synergistic effect of multiple action targets. Particularly importantly, the regulatory effect of prebiotics on the expression of clock genes may improve sleep problems by restoring circadian rhythms and has a potential improvement effect on the core symptoms of SCZ by regulating the balance of neurotransmitters. To promote the translational application of prebiotic therapy in the clinical practice of mental diseases, subsequent studies should further clarify the application values of different prebiotics, individualized dosing strategies, and the safety and effectiveness evaluations of continuous treatment.

### 5.3 Dietary interventions

Dietary adjustment is also an important component of personalized treatment. A lot of scientific evidence has demonstrated the relationship between diet and sleep. Consuming healthy foods promotes better sleep quality, while higher intakes of processed and free-sugar-rich foods are associated with poorer sleep characteristics.^103^ Studies have found that insomnia and sleep duration are genetically correlated with eating habits, and specific dietary components (such as fat and protein) and eating habits (such as beef, coffee, and dried fruit intake) can affect sleep through their interaction with gut microbiota.^104^ In recent years, the ketogenic diet has received extensive attention in the scientific community and the field of public health. Ketogenic diet intervention has been proven to be a feasible and acceptable adjuvant treatment method, improving both the mental and metabolic health of patients with mental disorders accompanied by metabolic abnormalities.^105^ The results of the Pittsburgh Sleep Quality Index (PSQI) assessment showed that the subjective sleep quality of patients with bipolar disorder and SCZ was improved after receiving ketogenic diet intervention.^106^ These results indicate that incorporating the ketogenic diet into the comprehensive treatment strategies for patients with mental disorders is expected to achieve multiple benefits in terms of mental symptom control, metabolic regulation, and sleep improvement.

In addition, from the perspective of a high-fiber diet, SCFAs are produced by the fermentation of dietary fiber by gut symbiotic bacteria. When dietary fiber intake is insufficient, the production level of SCFAs will be reduced, which will have an adverse impact on various physiological functions of the host.^107^ Increasing dietary fiber intake to boost SCFAs production can not only enhance the protective function of the blood‒brain barrier and maintain the integrity of the intestinal mucosa but also regulate the composition of gut microbiota, thus synergistically promoting the establishment of immune homeostasis.^108^ Based on this mechanism, this dietary intervention strategy provides new ideas for improving sleep in patients with mental disorders. By alleviating the intestinal inflammatory state and regulating neurotransmitter levels, it can improve patients' mental symptoms and sleep quality.

### 5.4 FMT

FMT, as a biotherapeutic strategy centered on microbial community reconstruction, involves transplanting fecal matter from a healthy donor into a patient's gastrointestinal tract. The core objective is to directly reconstitute the recipient's gut microbiota, promoting the restoration of a balanced microbial ecosystem and thereby achieving clinical therapeutic goals.^109^ As a breakthrough intervention method, it has shown unique advantages in animal models and preliminary clinical studies. By transplanting the gut microbiota of SCZ patients, SCZ-like behaviors, including hyperactivity, increased anxiety, impaired social interaction, and memory deficits, appeared in SPF mice.^110^ These mice also showed significant activation of the kynurenine-kynurenic acid pathway in peripheral and CNS tryptophan metabolism, along with enhanced extracellular dopaminergic neurotransmitter release in the basal cells of the prefrontal cortex, increased 5-HT levels in the hippocampus, accompanied by a decrease in the concentration of glutamatergic neurotransmitters in the hippocampus and an upregulation of glutamine and GABA levels.^111^^,^^112^

Similarly, FMT has also shown important value in human clinical studies. In the comparison of fecal samples before and after patients received FMT from healthy donors, a significant increase in alpha diversity was shown, as well as an increase in the abundances of Collinsella and *Bifidobacterium*.^113^ Paired t-tests and independent samples t-tests indicated that FMT treatment significantly reduced blood cortisol levels in patients, with corresponding improvements in sleep and mood-related assessment metrics.^114^ The promising evidence obtained from animal experiments and preliminary clinical trials highlights the translational value of research spanning from animal models to human studies. As a potential treatment intervention, FMT play a therapeutic role by regulating the composition and function of gut microbiota and then promoting the synthesis of neurotransmitters or their precursor substances.^115^ These studies indicate that by leveraging the complex interactions among the nervous, endocrine, and immune systems in the gut–brain axis, FMT can effectively regulate emotions and behaviors and has a positive promoting effect on alleviating the symptoms of patients with multiple mental disorders. While FMT holds broad therapeutic prospects, its clinical application still faces core challenges such as a lack of standardization, variable efficacy, and the need to verify long-term safety. In donor screening, detailed analysis of the gut microbiome using high-throughput sequencing technology can help effectively identify healthy and stable donors. Regarding treatment administration, oral FMT capsules offer greater convenience and improved patient compliance compared to traditional enema methods. Diverse delivery routes – such as colonoscopic infusion, nasoenteric tube administration, or oral capsules – can help balance efficacy with patient adherence. Establishing a follow-up safety monitoring system to prevent and control risks such as infection transmission will enhance safety oversight.^116^ Furthermore, when conducting FMT treatment, it is essential to pay close attention to the informed consent capacity of patients with mental disorders, ensuring they can make autonomous decisions based on a full understanding of the potential risks and benefits, and that applications are standardized through ethical review board approvals. Although more studies are still needed to verify the long-term effects of FMT in the treatment of SCZ and SD, it shows broad application prospects when it is part of personalized treatment programs.

### 5.5 Targeted metabolite drug treatments

In the aspect of precision medicine, targeted metabolite drug treatment is becoming a new research direction. Although traditional microbiota transplantation therapy has achieved certain results in clinical applications, the huge differences in the composition and function of gut microbiota among individuals have greatly limited the stability and repeatability of its treatment effects. However, directly supplementing microbial metabolites or their precursor substances provides a new idea for solving this problem. This strategy can bypass the uncertainties brought about by individual differences in the process of microbiota transplantation and directly act on the human metabolic network to achieve more precise regulation.^117^ SCFAs and the tryptophan metabolic pathway play a key role in human physiological and pathological processes. Based on this understanding, taking indoleamine 2,3-dioxygenase (IDO) inhibitors as an example, IDO is a key rate-limiting enzyme in the metabolism of tryptophan along the kynurenine pathway. Its excessive activation will lead to tryptophan depletion and the production of a series of neurotoxic metabolites, which will then trigger abnormal immune responses and neurological dysfunctions.^118^ IDO inhibitors can effectively regulate immune responses and reduce the production of neurotoxic metabolites by inhibiting the activity of IDO, showing great potential in the treatment of various diseases.^119^ As mentioned above, SCFAs and tryptophan metabolites are closely related to the pathogenesis of SCZ and sleep regulation. Future research should further explore the specific mechanism of action of metabolites and promote their development in clinical applications, which may provide safer and more effective treatment options for patients with SCZ combined with SD.

### 5.6 Potential therapeutic strategies

It is important to note that not all patients with SCZ are suitable candidates for microbiome-targeted interventions. Identifying potential candidates should be based on specific characteristics, such as the presence of clear gastrointestinal symptoms, poor response to or significant side effects from antipsychotic medications, a strong correlation between abnormal sleep architecture and disease activity, and microbiome profiling results indicating significant deviation from healthy controls in both composition and function of the gut microbiota. Target populations should be selected based on a combination of clinical phenotypes and microbial features. When advancing such microecological interventions, the priority of different strategies should be clearly defined. Low-risk, noninvasive approaches should be explored first. For example, supplementation with specific probiotics and prebiotics may serve as a foundational intervention; dietary pattern modification can be considered a long-term management strategy. In contrast, high-risk and less predictable therapies such as FMT should only be considered for patients with treatment-resistant symptoms, inadequate response to conventional therapies, and severe gut microbiota dysbiosis, under conditions of fully informed consent and strict ethical oversight. Furthermore, personalized differences and potential risks must be carefully considered during implementation. Probiotic and prebiotic interventions should account for strain-specific effects and individual tolerance. Dietary interventions need to consider patient adherence and nutritional balance. For FMT, rigorous donor screening is essential, along with close monitoring for infections, immune and metabolic adverse reactions, long-term psychiatric outcomes, and the establishment of a dynamic efficacy-safety evaluation system.

## 6. Conclusion and perspectives

SD accompanying SCZ is a complex clinical problem involving multiple systems and levels. This review systematically elaborates on the potential mechanism of action and treatment value of gut microbiota in this comorbid phenomenon by integrating existing evidence. Firstly, we analyzed the characteristic sleep manifestations of SCZ patients, including disrupted sleep continuity, reduced slow-wave sleep, shortened REM sleep latency, and other characteristic changes. It is worth noting that the abnormalities of these sleep parameters are not only related to the severity of the disease but may also be involved in the pathological process of psychotic symptoms. Our summary shows that the gut microbiota characteristics exhibited by SCZ patients are significantly similar to those in the studies of SD models. Through comparative analysis, it was found that both types of research results show characteristic changes such as a decrease in gut microbiota alpha diversity, a reduction in SCFAs-producing bacteria, and an increase in the proportion of proinflammatory microbiota. Therefore, we hypothesize that the prevalent gut microbiota dysbiosis in SCZ patients may be a significant factor contributing to decreased sleep quality, while sleep deterioration, in turn, exacerbates psychiatric symptoms through feedback mechanisms, thereby forming a vicious cycle. The breakthrough progress in the research on the MGBA in recent years has provided a new perspective for understanding this association. The gut microbiota affects the function of the CNS through the complex regulatory network mediated by the MGBA, and its mechanisms involve the regulation of neurotransmitter synthesis by microbial metabolites, neuroinflammation triggered by enterogenic immune responses, signal transduction mediated by the vagus nerve, and the impact on the integrity of the blood‒brain barrier and neuronal plasticity. This review revealed that in patients with SCZ, disruptions in the gut microbiota and the diurnal secretion of their metabolites (SCFAs and tryptophan derivatives) dynamically interact with the expression of core circadian clock genes (BMAL1/CLOCK), thereby disrupting the stability of the sleep‒wake cycle. Concurrently, gut dysbiosis can induce systemic low-grade inflammation, promoting the release of proinflammatory cytokines such as IL-6 and TNF-*α*, which not only affect the central nervous system but also directly interfere with normal sleep architecture. The persistent deterioration of sleep further exacerbates both gut microbiota imbalances and psychiatric symptoms through stress responses (HPA axis overactivation) and feedback via the vagus nerve pathway. Consequently, gut dysbiosis, central nervous system dysfunction, and disrupted sleep homeostasis form a self-perpetuating dynamic cycle within the host, leading to a periodic worsening of psychopathological manifestations and physiological rhythm disturbances. These findings provide an important theoretical framework for understanding the mechanisms underlying comorbid SD in SCZ patients. Targeting the gut microbiota to disrupt this vicious cycle offers a promising novel therapeutic perspective for such patients.

In terms of treatment, existing studies suggest that we need to adopt an integrated treatment strategy. Although atypical antipsychotic drugs can improve psychotic symptoms, their complex impact on sleep architecture requires more detailed evaluation. The improvement effect of CBT on insomnia is also effective in comorbid patients, but it needs to be appropriately adjusted according to psychotic symptoms. Therefore, intervention strategies targeting the gut microbiota are evolving towards a multilevel and precision-oriented approach. Specifically, supplementation with specific probiotics and prebiotics, along with dietary modifications, serve as low-risk foundational interventions that exert synergistic improvements on psychiatric symptoms and sleep quality through multitarget mechanisms. FMT, as a higher-level intervention with microbiota-remodeling potential, shows clinical promise, yet its definitive therapeutic role requires establishment through standardized safety and efficacy research. In terms of implementation, focus should be placed on patients with persistent gastrointestinal symptoms, inadequate response to or poor tolerance of medication, a high correlation between psychiatric symptom severity and sleep rhythm disruption, and those whose gut microbiota structure and functional profile significantly deviate from healthy references with a potential link to clinical manifestations. Typing models integrating clinical phenotypes and microbial characteristics will provide a scientific basis for precisely applying different intervention strategies, ultimately advancing microbiota-targeted therapies from generalized interventions towards systematic, personalized precision applications.

Despite the aforementioned progress, the field still faces several key challenges. There is a severe lack of high-quality clinical research focusing specifically on SCZ comorbid with SD, and even fewer studies analyzing the condition from a gut microbiota perspective. Existing studies predominantly rely on 16S rRNA sequencing, which, while effective for revealing community structural changes, has limitations in species resolution and functional inference, leading to variability between studies and difficulty in cross-validation. Metagenomics, as a key tool for in-depth mechanistic exploration, can directly obtain functional gene profiles and is expected to move beyond genus-level associations to provide more reliable evidence for elucidating causal mechanisms like SCFA metabolism and immune regulation. However, methodological differences are only one part of the challenge. The reproducibility of research in this field is severely tested by numerous factors. Firstly, sample sizes are generally insufficient, and studies are often single-center, limiting the generalizability of findings to specific populations and local environments. There is an urgent need for large-scale, multicenter collaborative studies to validate existing findings. Secondly, many studies fail to report detailed medication information of patients, and the inherent heterogeneity of the disease acts as a potential confounder, possibly introducing bias into the conclusions. Furthermore, a lack of standardization in experimental designs across studies, from sample collection and DNA extraction to bioinformatic analysis, directly hinders effective comparison and integration of results. To address this systemic challenge, promoting standardized experimental protocols and establishing open raw data platforms are crucial. This will facilitate independent verification and secondary analysis of data, thereby enhancing the robustness of the field's conclusions.

Future study can delve into the sleep variations among SCZ patients from a multiomics perspective, observing the gut microbial composition of SCZ patients with different sleep qualities. Starting from the interaction between the gut microbiota and metabolites, efforts should shift from descriptive research to mechanistic studies, aiming to elucidate the intrinsic mechanisms by which they affect host sleep quality. Through meticulous experimental design, technological innovation, and interdisciplinary collaboration, there is potential to move beyond correlations, establish causality, and thereby propel basic discoveries toward clinical diagnosis and treatment translation, ultimately providing a scientific basis and practical support for improving clinical outcomes in patients with this comorbidity.

## Disclosure of potential conflicts of interest

The authors declare no conflicts of interest.

## Data Availability

Not applicable.
